# Perceived need for substance use treatment among young women from disadvantaged communities in Cape Town, South Africa

**DOI:** 10.1186/1471-244X-14-100

**Published:** 2014-04-04

**Authors:** Bronwyn Myers, Tracy L Kline, Irene A Doherty, Tara Carney, Wendee M Wechsberg

**Affiliations:** 1 Alcohol and Drug Abuse Research Unit, South African Medical Research Council, Cape Town, South Africa; 2 Department of Psychiatry and Mental Health, University of Cape Town, Cape Town, South Africa; 3 RTI International, Research Triangle Park, Raleigh, North Carolina, USA; 4 Gillings Global School of Public Health, University of North Carolina, Chapel Hill, North Carolina, USA; 5 Psychology in the Public Interest, North Carolina State University, Raleigh, North Carolina, USA; 6 Psychiatry and Behavioral Sciences, Duke University School of Medicine, Raleigh, North Carolina, USA

**Keywords:** Perceived need for drug treatment, Mental health, Women, South Africa, Methamphetamine

## Abstract

**Background:**

Initiation of treatment for substance use disorders is low among young women from disadvantaged communities in Cape Town, South Africa. Yet little is known about the factors that influence perceived need for treatment (a determinant of treatment entry) within this population.

**Methods:**

Baseline data on 720 young, drug-using women, collected as part of a randomized field experiment were analyzed to identify predisposing, enabling and health need factors associated with perceived need for treatment.

**Results:**

Overall, 46.0% of our sample perceived a need for treatment. Of these participants, 92.4% wanted treatment for their substance use problems but only 50.1% knew where to access services. In multivariable logistic regression analyses, we found significant main effects for ethnicity (AOR = 1.54, 95% CI = 1.05-1.65), income (AOR = 0.96, 95% CI = 0.93-0.99), anxiety (AOR = 1.22, 95% CI = 1.05-1.45), and not having family members with drug problems (AOR = 1.45, 95% CI = 1.05-2.04) on perceived need for treatment. When the sample was stratified by methamphetamine use, income (AOR = 0.87, 95% CI = 0.79-0.96), awareness of treatment services (AOR =1.84, 95% CI = 1.03-3.27), anxiety (AOR =1.41, 95% CI = 1.06-1.87) and physical health status (AOR = 6.29, 95% CI = 1.56-25.64) were significantly associated with perceived need for treatment among those who were methamphetamine-negative. No variables were significantly associated with perceived need for treatment among participants who were methamphetamine-positive.

**Conclusions:**

A sizeable proportion of young women who could benefit from substance use treatment do not believe they need treatment, highlighting the need for interventions that enhance perceived need for treatment in this population. Findings also show that interventions that link women who perceive a need for treatment to service providers are needed. Such interventions should address barriers that limit young women’s use of services for substance use disorders.

## Background

In South Africa, the prevalence of substance use disorders is high [1,2], with the lifetime and past year prevalence of substance use disorders among adults being 13.3% and 5.8%, respectively [1]. Among these 12-month prevalent cases, approximately 30.9% have a severe substance use disorder and therefore are likely to benefit from treatment [1]. Epidemiological studies suggest that substance use disorders are concentrated in the Western Cape Province [2]. In this province, the lifetime prevalence of substance use disorders among the general population is significantly higher (20.3%) and problem severity greater than the national average [1]; suggesting a considerable need for substance use treatment services in this province. The widespread use of methamphetamine among people who use drugs in the Western Cape [2-4] is a likely contributing factor to the severity of substance use disorders in this province, as people who use methamphetamine are significantly more likely to report worse health and social problems than people who use other substances [5,6].

Even though evidence-based treatment programs are available in the Western Cape [7], only a small proportion of people with substance use disorders in this province ever receive treatment [8,9]. Women from disadvantaged communities are significantly less likely than men to obtain treatment for these disorders, even when the severity of their drug problems is comparable [5,10]. While systemic and structural barriers to accessing treatment have been identified among women from impoverished communities in the Western Cape [11,12], these barriers do not fully account for why poor substance-using women fail to utilize available services. Failure to perceive a need for treatment, a key determinant of health services use [13] and a necessary step in the process of changing addictive behaviors [14], may contribute to the poor uptake of treatment for substance use disorders by vulnerable women. Perceived need for treatment is often low among people with substance use disorders [15], including poor South African women [9,16,17]. One study reported that only 39.8% of women who met DSM-IV criteria for a current substance use disorder perceived a need for treatment [17]. As people who perceive a need for treatment are more likely to initiate treatment and have better substance use outcomes than those who do not think treatment is necessary [15,18-20], helping substance-using women recognize a need for treatment may be an important focus for interventions directed at improving treatment initiation among disadvantaged women.

Nationally representative surveys of substance use in the United States (US) have identified correlates of perceived need for treatment that deepen our understanding of the characteristics of people who may benefit from interventions to improve perceived need for treatment. These studies found that socio-demographic factors such ethnicity (not white) [15,21,22], marital status (being unmarried) [15,21,23], age (being older) [21], and education (higher education) [24] were associated with increased odds of perceiving a need for treatment. In addition, having a poorer physical [21,23] and mental health status [15,21-23], greater alcohol [15,22] and drug use [15,18] problem severity, greater functional and social impairment [18,23] and more emotional support [24] increased the likelihood of perceiving a need for treatment. People with stimulant use disorders were also more likely to perceive a need for treatment than people with other types of substance use disorders [15]. Finally, prior substance use treatment and having a family history of substance use disorders has also been positively associated with perceived need for substance use treatment [15,18,22].

While these studies provide insights into factors associated with perceived need for substance use treatment in the US, it is unclear whether these correlates are relevant for low- and middle-income countries, such as South Africa. Furthermore, research on this topic has largely examined correlates of perceived need for treatment among populations undifferentiated by gender, with no studies investigating factors associated with perceived need for treatment among women specifically. These knowledge gaps are cause for concern as they limit our ability to identify women who may benefit most from interventions that enhance perceived need for substance use treatment.

As a first step to addressing this gap, we explored the correlates of perceived need for substance use treatment among young, drug-using women from Cape Town, South Africa. The Behavioral Model of Health Services Utilization (BHSU) [13] was the theoretical basis for the study and guided variable selection. Although the BHSU was originally developed to understand health services utilization [8,13], it has also been used to predict perceived service need [25,26]. According to the BHSU, perceived need is a function of the separate and combined influence of predisposing factors (e.g. demographic, attitudinal-belief variables), enabling conditions that encourage the person to perceive a need for services (e.g. income, relationship status, awareness of services, and social support), and health needs (e.g. physical and mental health status and functional impairment) [13,25,26]. Using this framework, this study aimed to identify the profile of variables associated with perceived need for treatment among young substance-using women from disadvantaged communities in Cape Town, South Africa. As methamphetamine use is highly prevalent among young women from these communities [3,4] and because people who use methamphetamine report greater physical and mental health problems and less social support relative to people who use other types of substances [3-6], we hypothesized that there might be differences in the pattern of correlates associated with perceived need for treatment among young women with and without recent methamphetamine use. As a result, we also compared and contrasted the profile of variables associated with perceived need for treatment among young women with and without recent methamphetamine use.

## Methods

### Study design, participants and procedures

In this article, we report on the baseline characteristics of 720 women recruited into a community-based randomized field experiment of an HIV risk-reduction intervention for subtance-using women, known as the Western Cape Women’s Health CoOp (WHC). Participant recruitment started in September 2008 and 12-month follow-up ended in January 2012 [27].

Eligible participants were sexually active heterosexual women between 18 and 33 years of age, living in one of the study’s target communities, who had used at least two substances (one of which could be alcohol) at least once a week for the past three months, and who had not participated in the Women’s Health CoOp pilot or formative studies [28]. We decided to recruit women between 18 and 33 years of age as South African women in this age range are most-at-risk for acquiring HIV and we were interested in recruiting women who would benefit most from a targeted HIV prevention program [27]. To be selected as a target community, areas had to be located around Cape Town’s international airport and be recognized as a disadvantaged, low-income area by the provincial authority responsible for social welfare and community upliftment programs [29]. We used a rigorous sampling strategy in which all 15 disadvantaged communities in this region were selected as target communities. To ensure balanced recruitment, population estimates were used to calculate desired sampling targets for each of the selected areas [27].

As the target population for this study is generally difficult to reach, trained outreach workers used standard street outreach techniques (such as distributing marketing materials in places frequented by our target population and visiting these areas regularly to build trust and rapport with community members) to identify potential participants. Outreach workers were lay health workers who resided in similar communities, all of whom had experience in community-based recruitment for mental health studies. These staff approached adult women in the communities of interest, described the study, and requested verbal permission to screen them for potential study eligibility [4,27]. Once we had built trust and rapport with community members, we had little difficulty in recruiting women to participate in the study, largely because substance use is normalized and occurs in public spaces in many of the communities we recruited from [30]. Eligible women who were interested in the study were given an appointment for an intake interview and were also asked to refer women similar to themselves to the outreach workers for eligibility screening. At the intake appointment, women were rescreened and women who remained eligible were enrolled only after providing written informed consent to participate in the study. Once enrolled, an interviewer administered the baseline questionnaire by using computer-assisted personal interviewing (CAPI) and conducted biological testing for pregnancy, HIV, and recent drug use. All assessments occurred in English, with field staff able to translate questions where needed. Participants received refreshments and a grocery voucher valued at ZAR 40 (approximately $6 at the time of the study) for their time. Further detail on the design and procedures of this experiment are provided elsewhere [27].

### Measures

A modified Revised Risk Behavior Assessment (RRBA), adapted for use in the Western Cape [31], was used to collect baseline information. The RRBA collects self-report information on socio-demographic characteristics, health knowledge, substance use and readiness for treatment (including perceived need for drug treatment), sexual risk behaviors, power and empowerment, conflict and victimization, physical and mental health, and need for health and other services. For this paper, our outcome variable of interest was perceived need for drug treatment. To address our study aims, we explored associations between this outcome variable and various predisposing, enabling and health need variables.

#### Perceived need for drug treatment

The main outcome variable for this paper is perceived need for substance use treatment. Specifically, participants were asked about whether or not they thought they needed treatment now. This item had a yes/no response option and was part of an assessment of readiness to change. In addition, participants who perceived a need for treatment were asked whether they wanted to go to treatment. Those who wanted to go to treatment were asked whether they wanted to go to treatment within the next 30 days, between 30 days and six months or after more than six months.

#### Predisposing factors

Predisposing factors included race/ethnicity, specifically whether the participant self-identified as Black African, “Coloured”, White, or Asian/Indian; age; and education (whether or not the person had completed high school); all of which have been associated with perceived need for treatment in the literature. In South Africa, the term “Coloured” is an officially accepted descriptor of race/ethnicity and describes people of mixed race ancestry who belong to a unique ethnic group in South Africa with their own language and cultural traditions.

#### Enabling factors

Enabling factors included income in last 30 days (in ZAR) and relationship status. For relationship status, participants were asked to indicate whether or not they were single, were involved in a relationship but were not living with their partner, or were married and/or living with their partner. Other enabling factors of interest included whether the participant had ever received treatment for a substance use disorder whether the participant had family members with drug problems, and whether the participant was aware of where to go for substance use treatment. These items all had yes/no response options.

#### Health need variables

We examined physical and mental health status, both of which have been associated with perceived need for substance use treatment in the literature. Participants were asked to rate their current physical health status as poor or good. We also examined two aspects of psychological distress: depression and anxiety. We used the Center for Epidemiological Studies Depression (CES-D) scale to measure symptoms of depression. This scale consists of 20 items which are rated on a four-point Likert scale with responses ranging from 0 (No symptom presence) to 3 (Symptoms are present most or all of the time). Composite scale scores range from 0 to 60, with a score of 16 or higher signifying clinically meaningful depression [32]. Among different populations, the CES-D has shown acceptable psychometric properties [33] and has been validated for use in South Africa [34]. This study obtained a Cronbach’s alpha coefficient of 0.89 for the CES-D. We used the seven-item Texas Christian University (TCU) anxiety scale, purposefully developed for use among substance-using populations, to assess symptoms of anxiety. For this scale, items are rated on a five-point Likert scale, with responses ranging from 1 (Disagree strongly) to 5 (Agree strongly). Composite scores range from 7 to 35, with higher scores indicating more symptoms of anxiety. Prior research with substance-using populations [35,36], including South African drug using populations [8-11] has indicated that the TCU anxiety scale has good reliability and validity. This study obtained a Cronbach’s alpha coefficient of 0.80 for this scale.

#### Methamphetamine use

We further investigated how methamphetamine use related to perceived need for treatment. Recent drug use was assessed through biological testing. The Reditest drug test (Redwood Toxicology Laboratory) was used to test participants’ urine samples for the use of methamphetamine, cocaine, opiates, and THC (cannabis) in the past 72 hours. For this paper, we report on the biological results for methamphetamine use which were used to stratify the sample.

### Ethics statement

Ethics approval was granted by the Institutional Review Boards of RTI International and Stellenbosch University’s Faculty of Health Sciences. The trial (on which this study is based) is registered on ClinicalTrials.gov (Trial Registration Number: NCT00729391).

### Statistical analyses

Missing data was dealt with through list-wise deletion of cases with missing observations on our variables of interest. Our final analytical sample comprised 719 cases (of a possible 720). We performed tabular and other descriptive analyses on all data. Chi-square tests of association were used to examine associations between methamphetamine use and the categorical variables and independent sample t-tests were used to compare the methamphetamine use groups on continuous variables. Next, simple and multiple logistic regression analyses were conducted to examine unadjusted and adjusted associations between perceived need for treatment and various predisposing, enabling and health need variables for the overall sample. All covariates were entered into the multiple logistic regression analyses. As we were interested in whether the profile of factors associated with perceived need for drug treatment varied by subgroups of substance use, we then stratified the sample by recent methamphetamine use. The size of the associations between the covariates and perceived need for drug treatment were then re-estimated via separate multiple logistic regression analyses for the sub-samples of individuals who tested positive and negative for methamphetamine use. The results of these regression models were reported as odds ratios (ORs) with 95% confidence intervals (95% CI). All statistical analyses were conducted using SPSS 21 (Chicago, Il).

## Results

Of the 1098 women screened for eligibility, 247 did not meet the study’s inclusion criteria and another 131 did not wish to participate in the study. Women who did not want to participate reported that they did not have time to participate in the study, were concerned that fieldworkers were social workers and could remove children from their care, and felt that their drug use was not problematic.

### Sample description

The sample was comprised entirely of Black African (44.9%) and Coloured women (55.1%). Of the Coloured participants, 82.6% tested positive for recent methamphetamine use compared to 36.5% of Black African participants (p < 0.001). In addition, women who tested positive for methamphetamine were somewhat older (mean age of 23.5 versus 22.6 years, p = 0.004), had higher monthly incomes (R304.2 versus R211.4, p = 0.009) than women who tested negative for methamphetamine (Table 1). For the overall sample, the mean CES-D score was 26.0 (SD = 12.3), considerably higher than the cut-off score of 16 used to signify clinically meaningful depression [28]. The mean CES-D (p <0.001) and TCU anxiety (p <0.001) scores were significantly higher among women who tested positive versus those who tested negative for methamphetamine (Table 1). Overall, 12.4% of the sample reported poor physical health; women who tested positive for methamphetamine were more likely to report poor physical health than those who tested negative (p <0.001; Table 1).

**Table 1:** Perceived need for treatment and predisposing, enabling and health need variables for women with and without recent methamphetamine use

**Variable**	**Total**	**Methamphetamine positive (N =445)**	**Methamphetamine negative (N = 274)**	**χ**^**2**^**/*t*** **(df)**	***p***
***Predisposing variables***					
Age (M, SD)	23.1 (4.2)	23.5 (4.2)	22.6 (4.2)	2.9 (717)	0.004
Ethnicity (Coloured; %, n)	55.1% (445)	82.6% (327)	17.4% (69)	159.9 (1)	<0.0001
Education (not completed high school; %, n)	88.9% (639)	89.4% (398)	88.0% (241)	0.4 (1)	0.539
***Enabling variables***					
Income in the last 30 days (ZAR M, SD)	268.4 (464.0)	304.2 (493.5)	211.4 (406.7)	2.6 (717)	0.009
Relationship status (%, n)					
Married or living as married	21.7% (156)	27.6% (123)	12.0% (33)		
Boyfriend	73.9% (531)	66.5% (296)	85.8% (335)	32.61 (2)	<0.0001
Single	4.5% (32)	5.8% (26)	7.2% (6)		
Family members with drug problems (Yes; %, n)	57.4% (413)	61.1% (274)	51.5% (141)	6.8 (1)	0.011
Prior treatment (Yes; %, n)	7.4% (53)	10.1% (45)	2.9% (8)	12.8 (1)	<0.0001
Awareness of treatment programs (Yes, %, n)	50.1% (360)	52.9% (145)	48.3% (215)	1.44(1)	0.230
***Health need variables***					
CES-D (M, SD)	26.0 (12.3)	28.5 (11.9)	21.9 (11.8)	7.3 (717)	<0.0001
Physical health (Poor; %, n)	12.4% (89)	15.7% (70)	6.9% (19)	12.10 (1)	0.001
TCU anxiety (M, SD)	18.2 (6.1)	19.4 (5.8)	16.3 (6.0)	6.8 (717)	<0.0001
***Need and readiness for drug treatment***					
Perceived need for drug treatment (Yes; %, n)	46.0% (330)	46.3% (206)	45.4% (124)	0.5 (1)	0.820
Want to go to treatment (N = 329)	92.4% (304)	89.4% (110)	94.2% (194)	2.47 (1)	0.116
How soon do you want to go to treatment (N =304)					
Within next 30 days	63.8% (194)	63.4% (123)	64.5% (71)	0.18 (2)	
> 30 days < 6 months	24.0% (73)	24.7% (48)	22.7% (25)		0.915
> 6 months from now	12.2% (37)	11.9% (23)	12.7% (14)		

Less than half (46.0%) of the sample reported a need for drug treatment, with no significant differences found between women who tested positive versus those who tested negative for methamphetamine (Table 1). Of the participants who conveyed the need for treatment, 92.4% wanted to go to treatment. Of these, 63.8% wanted to go to treatment within the next 30 days (Table 1). Only half the sample (50.1%) knew where to go for drug treatment. More than half (57.4%) of the sample reported that they had family members with drug problems, with a greater proportion of women who tested positive for methamphetamine reporting family members (61.1%) with current drug problems than women who tested negative for this drug (51.5%; p = 0.011). Only a small proportion of participants reported prior treatment for a substance use disorder (7.4%). Compared to women without recent methamphetamine use, women with recent use were significantly more likely to have obtained prior treatment (p <0.0001; Table 1).

### Associations between predisposing, enabling and health need variables and perceived need for treatment

Results of the simple logistic regression models indicated two enabling variables associated with perceived need for drug treatment. For every R100 increase in past month income, the odds of perceiving a need for treatment decreased by 4% (OR = 0.96, 95% CI = 0.93-0.99; Table 2). Participants who reported not having family members with drug problems had 40% greater odds of perceiving a need for drug treatment than participants who had family members with drug problems (OR = 1.40, 95% CI = 1.04-1.88; Table 2). Health need variables were also associated with perceived need for treatment. Every five unit increase on the TCU anxiety scale (OR =1.20, 95% CI = 1.06-1.36) and the CES-D (OR =1.07, 95% CI = 1.01-1.13) increased the odds of perceiving a need for treatment (Table 2).

**Table 2:** Unadjusted and adjusted associations between predisposing, enabling and health need variables and perceived need for drug treatment among young, substance-using women in Cape Town, South Africa

**Covariates**	**Simple logistic regression**			**Multiple logistic regression**		
	**OR**^**a**^	**95% CI**^**b**^	***p***	**AOR**^**c**^	**95% CI**	***p***
***Predisposing variables***						
Age	0.97	0.74-1.88	0.108	0.98	0.94-1.01	0.188
Ethnicity (Black African)	1.21	0.90-1.62	0.212	1.54	1.05-1.65	0.027
Education (Not completed high school)	1.18	0.74-1.88	0.501	0.93	0.56-1.53	0.760
***Enabling variables***						
Relationship status (Reference married)						
Boyfriend	1.24	0.86-1.78	0.249	1.08	0.73-1.60	0.706
Single	1.22	0.57-2.62	0.607	1.10	0.50-2.42	0.819
Income in the last 30 days	0.96	0.93-0.99	0.015	0.96	0.93-0.99	0.041
Family with drug problems (No)	1.40	1.04-1.88	0.027	1.45	1.05-2.04	0.025
Prior treatment (No)	0.81	0.46-1.42	0.480	0.78	0.42-1.43	0.415
Awareness of treatment services (Yes)	1.27	0.95-1.71	0.109	1.19	0.86-1.65	0.301
***Health need variables***						
CES-D	1.07	1.01-1.13	0.034	1.01	0.99-1.03	0.159
TCU anxiety	1.20	1.06-1.36	0.003	1.22	1.05-1.45	0.017
Physical health status (Good)	0.66	0.42-1.03	0.069	0.75	0.46-1.22	0.245

^a^OR = odds ratio.

^b^95% CI = 95% confidence intervals.

^c^AOR = adjusted odds ratio. All predisposing, enabling and health need variables were entered into the multiple logistic regression model.

After all covariates were entered into the logistic regression model, anxiety (AOR = 1.22, 95% CI = 1.05-1.45), not having family members with drug problems (AOR = 1.45, 95% CI = 1.05-2.04), and income (AOR = 0.96, 95% CI = 0.93-0.99) remained significantly associated with perceived need for treatment (Table 2). Race/Ethnicity was also associated with perceived need for treatment. Specifically, Black African participants had 54% greater odds of perceiving a need for drug treatment than their Coloured counterparts (AOR = 1.54, 95% CI = 1.05-1.65; Table 2).

When we stratified the sample by methamphetamine use, having family members with drug problems was no longer significantly associated with perceived need for treatment (Table 3). Among participants who tested negative for methamphetamine, every R100 increase in past month income decreased the odds of perceiving a need for treatment by 13% (AOR = 0.87, 95% CI = 0.79-0.96; Table 3). Women who were aware of where to go for treatment had almost double the odds of perceiving a need for treatment relative to those who were unaware of services (AOR = 1.84, 95% CI 1.03-3.27). The health need variables of physical health status and anxiety were also significantly associated with perceived treatment need (Table 3). With every five unit increase in the TCU anxiety scale, the odds of perceiving a need for treatment increased 1.41 times (95% CI = 1.06-1.87; Table 3). Finally, the odds of perceiving a need for treatment were six-fold greater among participants who reported poor health compared to those who reported ill health (AOR = 6.29, 95% CI = 1.56-25.64; Table 3). No variables were significantly associated with perceived need for treatment among participants with recent methamphetamine use (Table 3).

**Table 3:** Adjusted associations between predisposing, enabling and health need variables and perceived need for treatment among young substance-using women in Cape Town, South Africa; stratified by methamphetamine use

	**Methamphetamine positive**			**Methamphetamine negative**		
	**N = 445**			**N = 274**		
**Covariates**	**AOR**^**a**^	**95% CI**^**b**^	***p***	**AOR**	**95% CI**	***p***
***Predisposing variables***						
Age	1.00	0.96-1.05	0.873	0.93	0.87-1.01	0.058
Ethnicity (Black African)	1.48	0.89-2.46	0.131	1.96	0.90-4.29	0.091
Education (Not completed high school)	0.87	0.46-1.67	0.681	0.98	0.41-2.31	0.959
***Enabling variables***						
Relationship status (Reference married)						
Boyfriend	1.32	0.84-2.07	0.224	0.48	0.19-1.20	0.116
Single	1.36	0.57-3.26	0.486	0.30	0.03-2.67	0.282
Income in the last 30 days	0.98	0.94-1.02	0.378	0.95	0.79-0.96	0.039
Family with drug problems (No)	0.66	0.43-1.01	0.056	0.79	0.45-1.39	0.407
Prior treatment (No)	0.72	0.37-1.34	0.328	1.97	0.34-11.27	0.448
Awareness of treatment (Yes)	0.98	0.65-1.48	0.924	1.84	1.03-3.27	0.039
***Health need variables***						
CES-D	1.28	0.92-1.15	0.626	1.14	0.99-1.33	0.079
TCU anxiety	1.08	0.86-1.34	0.517	1.41	1.06-1.87	0.020
Physical health (poor)	1.07	0.62-1.85	0.812	6.29	1.56-25.64	0.010

^a^AOR = adjusted odds ratio. All predisposing, enabling and health need variables were entered into the multiple logistic regression models.

^b^95% CI = 95% confidence intervals.

## Discussion

This study is among the first to explore factors associated with perceived need for treatment among young, substance-using women in a low- and middle-income country. Unlike US studies that indicate only 11% to 15% of individuals who should obtain substance use treatment are likely to perceive a need for treatment [15,19-22], 46% of participants in this study conveyed a need for substance use treatment. This considerably higher level of perceived need for treatment is probably because all participants were enrolled in an integrated substance use and HIV risk reduction intervention and as a result may have been motivated to address their drug use. Nonetheless, a considerable proportion of participants with substance use problems did not think they needed treatment. As perceived need is a critical step in help-seeking for substance use disorders [12,13,20] and because it is necessary for positive treatment outcomes [18-20], these findings suggest that a substantial proportion of substance-using women from disadvantaged communities in Cape Town, South Africa may possibly benefit from brief interventions that build readiness to change and enhance perceived need for treatment.

In keeping with earlier studies [14,20-22] and with the BHSU theoretical framework [13], various health need variables were associated with perceived need for treatment among young women. Psychological distress increased the likelihood of young women conveying the need for substance use treatment. However, when the sample was stratified by methamphetamine use, psychological distress was only significantly associated with perceived need for treatment among participants who tested negative for methamphetamine use. Similarly poor physical health was only significantly associated with perceived need for treatment among participants with recent methamphetamine use. These findings were unexpected as participants who tested positive for methamphetamine had significantly more symptoms of depression and anxiety and were more likely to report poorer physical health than participants without recent methamphetamine use. These findings are in keeping with a large body of research which has demonstrated that people who use methamphetamine have poorer physical and mental health outcomes relative to people who use other types of drugs [4,6,18,37,38]. Among the enabling variables, income was significantly (albeit weakly) associated with perceived need for treatment for the overall sample, with the likelihood of recognizing a need for treatment increasing as income decreased. This is probably because income is a proxy indicator for socioeconomic stability [17,39] and socioeconomic instability related to substance use may lead to help-seeking [17,39]. Yet when the sample was stratified by methamphetamine use, this variable was only associated with treatment need among participants who tested negative for methamphetamine. These findings suggest that, at least for women without recent methamphetamine use, brief interventions to enhance perceived need for drug treatment should educate women about the negative health and socioeconomic consequences of continued drug use and how treatment can improve psychosocial functioning. Such interventions could potentially enhance women’s perceived need for treatment, although this hypothesis requires testing in a randomized controlled trial.

There are several possible explanations for why these health need and socioeconomic factors were not associated with perceived need for treatment among methamphetamine-positive women. First, it is possible that women who use methamphetamine interpret symptoms of mental illness and physical ill-health as short-term consequences of methamphetamine intoxication and withdrawal, and therefore might not find these symptoms as distressing or as compelling to address as women who use other drugs. Another possibility is that they may feel that methamphetamine helps them with cope with life stressors and psychiatric problems [30], thereby discounting the need for treatment. Third, the neurocognitive effects of methamphetamine [40] may limit women’s insight into how methamphetamine impairs psychosocial functioning. This in turn may negatively influence decision-making about the need for substance use treatment [39]. Regardless of the reason for these findings, our failure to find variables associated with perceived need for treatment among women who use methamphetamine point to the need for additional research in this population. In particular, qualitative research is needed to deepen our comprehension of the factors associated with perceived need for drug treatment among young women who use methamphetamine and how physical and mental health status impact on perceived need for drug treatment in the context of methamphetamine use. In addition, quantitative research is needed to explore whether cognitive deficits among methamphetamine users impact on perceptions of functional psychosocial impairment and perceived need for treatment.

In addition, having family members with drug problems decreased the likelihood of perceiving a need for treatment among the overall sample. Having family members with drug problems might normalize drug use within the family system, thereby diminishing individuals’ awareness of their own problematic drug use [39] and reducing the likelihood that family members will encourage and support help seeking for drug use [41]. As previous studies have noted that a lack of emotional and tangible social support for treatment-seeking impacts negatively on treatment initiation [11,30,42], interventions that provide women with emotional support for changing their drug use and tangible support (such as assisting with childcare and providing transport to appointments) could potentially address barriers to treatment entry [11] and enhance women’s perceived need for drug treatment, although this hypothesis requires testing.

Finally, a large proportion of our sample recognized that they needed treatment, indicating substantial unmet treatment need among young women from disadvantaged communities in Cape Town. Almost all of these participants wanted to go to treatment, with the majority willing to enter treatment within the next 30 days. However, close to half the sample were unaware of where they could obtain treatment. As awareness of treatment programs was associated with perceived need for treatment among women who were methamphetamine-negative, treatment programs should consider ways in which they can improve their visibility within disadvantaged communities. Programs that conduct community-based outreach and screening to identify women who perceive a need for drug treatment and to link them to care may be helpful in addressing this treatment gap.

To successfully link young substance-using women to treatment, more work needs to be done to ensure that available services are a good fit for this underserved population. In this study, prior treatment for a substance use disorder was not significantly associated with perceived need for treatment, even though this variable has been consistently associated with need for treatment in the USA [15,18,22]. This suggests that young women who had previously obtained treatment may not have experienced prior treatment as sufficiently helpful to perceive a need for these services again, despite returning to substance use. This is not surprising as earlier research has shown that women from disadvantaged communities in Cape Town have some concerns about the appropriateness and effectiveness of substance use treatment that limit treatment seeking [11,12]. Efforts to link young substance-using women to treatment therefore should engage young substance-using women in discussions about their treatment and other service needs and perceptions of what constitutes acceptable and appropriate treatment. Information generated from these discussions may guide treatment providers on how to improve the fit of their services for young women.

Interpretation of these findings should however be considered in the light of some limitations. First, this paper was based on baseline data from a larger substance use and HIV risk reduction study. The strict inclusion and exclusion criteria of this randomized field experiment may have biased our ability to recruit a representative sample of substance-using women from poor communities in Cape Town. Our sample is likely biased towards women who do perceive their drug use to be problematic as some women who were eligible for this study declined to enroll as they did not see the need for an intervention. While this bias might have led to an over-reporting of perceived need for treatment, the main aim of this study was to examine correlates of perceived need rather than assess the prevalence of perceived need for treatment among this underserved population. Related to this, our choice of variables for these analyses was limited to those included in the larger experiment. As such, we were unable to explore potential associations between substance use severity, functional impairment and social support on perceived need for drug treatment. Future studies should consider including a broader range of covariates, including covariates that may be specific to the experiences of women such as number of dependent children, availability of child care and quality of relationship with partner. Third, as the sample was recruited from poor communities within Cape Town, the extent to which findings can be generalized to other parts of the province or country is unclear. Finally, the cross-sectional nature of the data makes it difficult to disentangle whether psychological distress and socioeconomic factors preceded or followed problematic substance use. Either could impact perceptions of need for treatment. To address this limitation, future research should consider prospective designs that enable researchers to unpack the temporal associations between variables associated with perceived need for treatment and explore whether perceived need for treatment predicts treatment initiation. These prospective studies will also be able to shed light on the temporal stability of perceived need for treatment, an area that has been under-researched.

## Conclusions

This study shows that a considerable proportion of young women who could potentially benefit from treatment for substance use disorders do not think they need treatment; highlighting the need for interventions that enhance perceived need for treatment among young women from poor communities in Cape Town, South Africa. Findings also reflect the need for interventions that link women who perceive a need for treatment to care. Such interventions should address the informational barriers that hamper treatment uptake as well as develop organizational readiness to provide appropriate and acceptable services to women with multiple service needs. A multi-pronged approach to improving treatment initiation among this vulnerable and underserved population is essential for reducing the public health burden of untreated substance use disorders in the region.

## Competing interests

The authors declare that they have no competing interests.

## Authors’ contributions

BM was a co-investigator on the study and was responsible for planning the paper, conducting the analyses and writing and reviewing all aspects of the manuscript. TLK and IAD assisted with the statistical analyses and writing the methods and results. WMW, TC and TLK and IAD reviewed the draft manuscript and provided critical comments and approved the final draft. WMW is the principal investigator for this project and senior author on this paper. She takes responsibility for the integrity of the data and the accuracy of the data analysis. All authors read and approved the final manuscript.

## Pre-publication history

The pre-publication history for this paper can be accessed here:

http://www.biomedcentral.com/1471-244X/14/100/prepub

## References

[B1] Herman AA Stein DJ Seedat S Heeringa SG Moomal H Williams DR The South African Stress and Health (SASH) study: 12-month and lifetime prevalence of common mental disorders S Afr Med J 2009 99 339 344 19588796 PMC3191537

[B2] Pasche S Myers B Substance misuse trends in South Africa Hum Psychopharmacol 2012 27 338 341 10.1002/hup.2228 22585594

[B3] Parry CDH Plüddemann A Myers B Wechsberg WM Flisher AJ Methamphetamine and sexual risk behaviour in Cape Town, South Africa: a review of 8 studies conducted between 2004 and 2007 Afr J Psychiatry 2011 14 372 376 10.4314/ajpsy.v14i5.4 22183467

[B4] Wechsberg WM Myers B Kline TL Carney T Browne FA Novak SP The relationship of alcohol and other drug use typologies to sex risk behaviors among vulnerable women in Cape Town, South Africa J AIDS Clin Res 2012 S1 15 015 23403403 10.4172/2155-6113.S1-015 PMC3568528

[B5] Burnhams NH Dada S Myers B Social service offices as a point of entry into substance abuse treatment for poor South Africans Subst Abuse Treat Prev Policy 2012 7 22 doi:10.1186/1747-597X-7-22 10.1186/1747-597X-7-22 22642796 PMC3414793

[B6] Plüddemann A Flisher AJ McKetin R Parry C Lombard C Methamphetamine use, aggressive behavior and other mental health issues among high school students in Cape Town Drug Alcohol Depend 2011 109 14 19 20064699 10.1016/j.drugalcdep.2009.11.021 PMC3784347

[B7] Temmingh H Myers B Ellis G, Stein D, Meintjies E, Thomas K Clinical treatment of substance use disorders in South Africa Substance Use and Abuse in South Africa: Brain, Behavioural and Other Perspectives 2012 Cape Town: University of Cape Town Press 329 366

[B8] Myers B Louw J Pasche S Inequitable access to substance abuse treatment services in Cape Town, South Africa Subst Abuse Treat Prev Policy 2010 5 28 doi:10.1186/1747-597X-5-28 10.1186/1747-597X-5-28 21073759 PMC2992042

[B9] Myers B Louw J Fakier N Alcohol and drug abuse: removing structural barriers to treatment for historically disadvantaged communities in Cape Town Int J Soc Welf 2008 17 156 165 10.1111/j.1468-2397.2007.00546.x

[B10] Myers B Vythylingum B Ellis G, Stein D, Meintjies E, Thomas K Women and alcohol Substance Use and Abuse in South Africa: Brain, Behavioural and Other Perspectives 2012 Cape Town: University of Cape Town Press 71 86

[B11] Myers B Louw J Pasche SE Gendered barriers to substance abuse treatment utilization among disadvantaged communities in Cape Town, South Africa Afr J Psychiatry 2011 14 146 153 10.4314/ajpsy.v14i2.7 21687914

[B12] Myers B Fakier N Louw J Stigma, treatment beliefs, and substance abuse treatment use in historically disadvantaged communities Afr J Psychiatry 2009 12 218 222 10.4314/ajpsy.v12i3.48497 19750251

[B13] Andersen RM Revisiting the behavioral model and access to medical care. Does it matter? J Health Soc Behav 1995 36 1 10 10.2307/2137284 7738325

[B14] DiClemente CC Schlundt D Gemmell L Readiness and stages of change in addiction treatment Am J Addict 2004 13 103 119 10.1080/10550490490435777 15204662

[B15] Hedden SL Gfroerer JC Correlates of perceiving a need for treatment among adults with substance use disorder. Results from a national survey Addict Behav 2011 36 1213 1222 10.1016/j.addbeh.2011.07.026 21855225

[B16] Jones HE Browne FA Myers BJ Carney T Ellerson RM Kline TL Poulton W Zule WA Wechsberg WM Pregnant and non-pregnant women in Cape Town, South Africa: drug use, sexual behaviour and the need for comprehensive services Int J Pediatrics 2011 article ID 353410, 8 pages 10.1155/2011/353410 PMC3083847 21541067

[B17] Wechsberg WM Wu L Zule WA Parry CD Browne FA Luseno WK Kline T Gentry A Substance abuse, treatment needs and access among female sex workers and non-sex workers in Pretoria Subst Abuse Treat Prev Policy 2009 4 11 doi:10.1186/1747-597X-4-11 10.1186/1747-597X-4-11 19473505 PMC2693506

[B18] Falck RS Wang J Carlson RG Krishnan LL Leukefeld C Booth BM Perceived need for substance abuse treatment among illicit stimulant drug users in rural areas of Ohio, Arkansas, and Kentucky Drug Alcohol Depend 2007 91 107 114 10.1016/j.drugalcdep.2007.05.015 17604917 PMC2679091

[B19] Kertesz SG Larson MJ Cheng DM Tucker JA Winter M Mullins A Saitz R Samet JH Need and non-need factors associated with addiction treatment utilization in a cohort of homeless and housed urban poor Med Care 2006 44 225 233 10.1097/01.mlr.0000199649.19464.8f 16501393

[B20] Mojtabai R Crum RM Perceived unmet need for alcohol and drug use treatments and future use of services: Results from a longitudinal study Drug Alcohol Depend 2013 127 59 64 10.1016/j.drugalcdep.2012.06.012 22770461 PMC3488160

[B21] Edlund MJ Booth BM Feldman ZL Perceived need for treatment for alcohol use disorders: Results from two national surveys Psychiatr Serv 2009 60 1618 1628 19952152 10.1176/appi.ps.60.12.1618 PMC2859201

[B22] Grella CE Karno MP Warda US Moore AA Niv N Perceptions of need and help received for substance dependence in a national probability survey Psychiatr Serv 2009 60 1068 1074 19648194 10.1176/appi.ps.60.8.1068 PMC2848480

[B23] Mojtabai R Olfson M Mechanic D Perceived need and help-seeking in adults with mood, anxiety, or substance use disorders Arch Gen Psychiatry 2002 59 77 84 10.1001/archpsyc.59.1.77 11779286

[B24] Edlund MJ Unutzer J Curran GM Perceived need for alcohol, drug and mental health treatment Soc Psychiatry Psychiatr Epidemiol 2006 41 480 487 10.1007/s00127-006-0047-1 16565918

[B25] Calsyn RJ Winter JP Predicting four types of service needs in older adults Eval Program Plann 2001 24 157 166 10.1016/S0149-7189(01)00006-4

[B26] Lundgren N Oral health and self-perceived oral treatment need of adults in Sweden Swed Dent J 2012 223 10 76 22519148

[B27] Wechsberg W Jewkes R Novak SP Kline T Myers B Browne FA Carney T Lopez AAM Parry C A brief intervention for drug use, sexual risk behaviours and violence prevention with vulnerable women in South Africa: a randomized trial of the Women’s Health CoOp BMJ Open 2013 3 e002622 doi:10.1136/BMJopen-002622 23793683 10.1136/bmjopen-2013-002622 PMC3657672

[B28] Wechsberg WM Karg RS Luseno W Young S Myers B Parry CDH Alcohol, cannabis, and methamphetamine use and other risk behaviors among black and Coloured South African women: a small randomised trial in the Western Cape Int J Drug Policy 2008 19 130 139 10.1016/j.drugpo.2007.11.018 18207723 PMC2435299

[B29] Smith K Cape Town 2025. The Status of Cape Town: Development Overview Report 2005 Cape Town: Isandla Institute http://isandla.org.za/publications/88/. Accessed 2013/October 9

[B30] Wechsberg W Myers B Reed E Carney T Emanuel AN Browne FA Substance use, gender inequity, violence and sexual risk among couples in Cape Town Cult Health Sex 2013 15 1221 1236 10.1080/13691058.2013.815366 23927691 PMC3818292

[B31] Wechsberg WM Revised Risk Behavior Assessment (RBA), Part I and Part II 1998 North Carolina, USA: Research Triangle Institute

[B32] Radloff LS The CES-D scale. A self-report depression scale for research in the general population Applied Psychological Measurement 1977 1 385 401 10.1177/014662167700100306

[B33] Naughton MJ Wiklund I A critical review of dimension-specific measures of health-related quality of life in cross-cultural research Qual Life Res 1993 2 397 432 10.1007/BF00422216 8161976

[B34] Hamad R Fernald LCH Karlan DS Zinman J Social and economic correlates of depressive symptoms and perceived stress in South African adults J Epidemiol Community Health 2008 62 538 544 10.1136/jech.2007.066191 18477753

[B35] Knight K Holcom M Simpson DD TCU Psychological Functioning and Motivation Scales. Manual on Psychometric Properties 1994 http://www.ibr.tcu.edu/pubs/datacoll/kk6-srf-95.pdf. Accessed 2013/October 9

[B36] Simpson DD Knight K Ray S Psychosocial and cognitive correlates of AIDS risky behaviors AIDS Education and Prevention 1993 5 121 130 8323854

[B37] Sutcliffe CG German D Sirirojn B Latkin C Aramrattana A Sherman SG Celentano DD Patterns of methamphetamine use and symptoms of depression among young adults in northern Thailand Drug Alcohol Depend 2011 101 146 151 19153017 10.1016/j.drugalcdep.2008.11.014 PMC2692876

[B38] Akindipe T Wilson D Stein DJ Psychiatric disorder sin individuals with methamphetamine dependence: prevalence and risk factors Metabolic Brain Disease 2014 doi:10.1007/s11011-014-9496-5 10.1007/s11011-014-9496-5 24532047

[B39] Quinn B Smoove M Papanastasiou C Dietze P An exploration of self-perceived non-problematic use as a barrier to professional support for methamphetamine users Int J Drug Policy 2013 24 619 623 10.1016/j.drugpo.2013.05.015 23867050

[B40] Scott JC Woods SP Matt GE Meyer RA Heaton RK Atkinson JH Grant I Neurocognitive effects of methamphetamine: a critical review and analysis Neuropsychol Rev 2007 17 275 297 10.1007/s11065-007-9031-0 17694436

[B41] EnglandKennedy ES Horton S Everything that I thought that they would be, they weren’t” Family systems as support and impediment to recovery Soc Sci Med 2011 73 1222 1229 10.1016/j.socscimed.2011.07.006 21880408 PMC3489269

[B42] Reed E Emanuael AN Myers B Johnson K Wechsberg W The relevance of social contexts and social action in reducing substance use and victimization among women participating in an HIV prevention intervention in Cape Town, South Africa Subst Abuse Rehabil 2013 4 55 64 24648788 10.2147/SAR.S45961 PMC3931639

